# Pilot evaluation of an objective structured assessment of technical skills tool for chest tube insertion

**DOI:** 10.3205/zma001194

**Published:** 2018-11-15

**Authors:** Mirco Friedrich, Julian Ober, Patrick Haubruck, Christian Bergdolt, Thomas Bruckner, Karl-Friedrich Kowalewski, Martina Kadmon, Beat-Peter Müller-Stich, Michael Christopher Tanner, Felix Nickel

**Affiliations:** 1 University of Heidelberg, Department of General, Visceral, and Transplantation Surgery, Heidelberg, Germany; 2 University of Heidelberg, HTRG – Heidelberg Trauma Research Group, Center for Orthopedics, Trauma Surgery and Spinal Cord Injury, Trauma and Reconstructive Surgery, Heidelberg, Germany; 3 University of Heidelberg, Institute for Medical Biometry and Informatics, Heidelberg, Germany; 4 University of Augsburg, Medical Faculty, Augsburg, Germany

**Keywords:** chest tube insertion, education, training, assesssment, hematothorax, pneumothorax

## Abstract

**Background: **Chest tube insertion is a standard intervention for management of various injuries of the thorax. Efficient clinical training of this and similar bed-side procedures is equally demanded and improvable. Here, we propose a nouveau means of assessment and feedback using an Objective Structured Assessment of Technical Skills (OSATS) tool. The modified OSATS for chest drain insertion is evaluated in a pilot trial focusing on chest drain insertion.

**Methods: **Participants in the pilot trial were medical students (3^rd^-6^th^ year of studies, n=9), junior residents (1^st^-3^rd^ post-graduate year, n=12), senior residents (4^th^-6^th^ post-graduate year, n=14), and attending surgeons (n=6) from Heidelberg University. Chest drain insertions on a cadaveric porcine model were rated by experts with the modified OSATS score. Participants’ performances were videotaped and subsequently rated by two remote experts (video rating). Primary aim was to assess criterion validity of the OSATS to distinguish experience levels.

**Results: **Kruskal-Wallis test showed significant differences between means of scores between four groups stratified by previous experience in chest tube insertion (level 0: 22.1±3.2 vs. level 1: 26.8±2.8 vs. level 2: 35.4±2.2 vs. level 3: 41.0±2.0; p=0.002; p_1,3_=0.049, p_0,3_=0.005). However, if groups were stratified by formal professional level, no statistically significant distinction could be made using OSATS. Hence, the OSATS tool showed criterion validity for differentiation between experience levels.

**Conclusion: **In the pilot study, the modified OSATS for chest tube insertion was apt to standardize expert rating and could be used to measure skill and to depict different experience levels. The OSATS will help facilitate training and assessment of chest drain insertion and could therefore improve surgical training for trauma situations. According to our data, the OSATS might be integrated into modern curricula.

## Zusammenfassung

**Hintergrund: **Die Thoraxdrainage ist ein etabliertes Standardverfahren zur Versorgung von Verletzungen des Thorax und der Lunge. Die effektive und zügige Anlage einer Thoraxdrainage setzt profunde Kenntnisse des Eingriffs voraus. Ein regelmäßiges Training sowie ein standardisiertes, objektives Feedback sind essentielle Voraussetzungen zur Sicherstellung einer optimalen Thoraxdrainagenanlage, auch unter Stresssituationen. Eine Möglichkeit zur Objektivierung und Strukturierung von Feedback besteht in der Anwendung von Assessment-Instrumenten wie dem Objective Structured Assessment of Technical Skill (OSATS). Ziel der vorliegenden Pilotstudie war die Evaluation eines modifizierten OSATS zur Thoraxdrainagenanlage hinsichtlich der Kriteriumsvalidität.

**Material und Methodik: **In die vorliegende Pilotstudie wurden 41 Probanden eingeschlossen (Medizinstudierende (3.-6. Studienjahr) n=9, Assistenzärzte (1.-3. Ausbildungsjahr) n=12, Assistenzärzte (4.-6. Ausbildungsjahr) n=14, Fachärzte n=6. Die Stratifizierung der Probanden erfolgte nach formalem Ausbildungsstand sowie nach individueller Vorerfahrung im Bereich der Thoraxdrainagenanlage in jeweils 4 Gruppen (Level 0-3). Die Thoraxdrainagenanlage erfolgte an Schweinekadavern unter standardisierten Bedingungen. Die Bewertung der Trainingsperformance erfolgte mittels Videoaufzeichnungen des Eingriffs (Videorating) durch zwei unabhängige Rater anhand des modifiziertem OSATS zur Thoraxdrainagenanlage.

**Ergebnisse: **Bei Stratifizierung nach Vorerfahrung der Probanden zeigten sich signifikante Unterschiede zwischen den Leistungen der vier Gruppen (Level 0: 22,1±3,2 vs. Level 1: 26,8±2,8 vs. Level 2: 35,4±2,2 vs. Level 3: 41.0±2,0; p=0.002; p_0,3_=0.005, p_1,3_=0.049,). Bei Stratifizierung nach formalem Ausbildungsstand zeigten sich hingegen keine signifikanten Unterschiede.

**Schlussfolgerung: **Die Ergebnisse der Pilotstudie zeigen die Kriteriumsvalidität des OSATS aufgrund dessen Fähigkeit zur Diskriminierung verschiedener Erfahrungslevel. Weiterhin ermöglicht die Anwendung des OSATS durch standardisierte und objektive Bewertung möglicherweise eine Verbesserung der Ausbildung im Bereich der Thoraxdrainagenanlage und leistet so einen Beitrag zur Optimierung von Handlungsabläufen im Notfallmanagement von Lungen- und Thoraxverletzungen. Basierend auf unseren Ergebnissen scheint eine Integration dieses OSATS in moderne Ausbildungskonzepte sinnvoll.

## Background

In many countries worldwide, consequences of demographic change affect the health care systems. A rising demand for medical assistance is opposing an equally rising physician shortage and distribution problem respectively [1], [2]. With facing such circumstances, optimization of processes within the health care system already has to take place in medical education and -training. Regarding that, Halsted’s concept of ‘see one, do one teach one’ is no longer feasible nowadays [3]. Modern medical training curricula need to be most efficient and effective as well, as costs and time consumption are factors commonplace to today’s clinical setting. Regarding those challenging circumstances there is an increasing need for frameworks and clear defined and standardized learning goals in medical education [4], [5], [6], [7]. This will help to develop competency based learning and teaching models [7] which could further build a framework of specific medical training curricula [7]. Consequently, many centers and universities worldwide provide specific procedural courses and research is conducted to optimize training [8], [9]. Aghdasi et al. adduced procedural training and accurate assessment of the technical skills of surgeons as an instance of such challenges. Generally carried out by experts in their field, i.e. senior surgeons, postgraduate training is currently time-consuming and costly but not replaceable, provided that validated training methods and assessment tools such as the Objective Structured Assessment of Technical Skills (OSATS) or the Global Operative Assessment of Laparoscopic Skills (GOALS) are deployed [10], [11], [12]. 

With increasing demand for partially invasive bedside procedures, adequate training capacities are needed to ensure each trainee’s skills are adequate prior to patient contact. This is supported by a needs assessment of Weitz et al., where physicians emphasized direct bed-side-skills as pivotal elements of medical education [13].

Training needs to be most efficient and effective as well, as costs and time consumption are factors commonplace to today’s clinical setting 

Chest tube insertion is an established minimally-invasive surgical intervention in treatment of acute trauma patients bearing injuries of lungs and thorax. Correct chest tube insertion facilitates efficient therapy without time loss or further complications that are potentially lethal due to the intervention’s vicinity to vital organs [14]. Thus, there is a need for a comprehensive assessment tool for training prior to the emergency situation that enables both versed physicians and novices to acquire the specific feedback necessary for proficiency in chest tube insertion. Early and decisive training using a tool as such might standardize handling of these emergency situations and could translate to improved trauma care and outcome. Assessment and feedback has been shown to be a valuable asset to surgical training [15]. In order to advance and standardize training success without straining staff and resources, we propose a nouveau Objective Structured Assessment of Technical Skill (OSATS) (see Table 1 (Tab. 1)) for chest tube insertion to standardize expert rating [16]. The aim of this manuscript was to evaluate the proposed OSATS score regarding its criterion validity by means of a pilot study. Therefore, the endpoint of the current pilot trial was the difference between means of scores obtained by groups differing in procedural expertise for blinded video rating.

## Material and Methods

### Evaluation of an Objective Structured Assessment of Technical Skills for chest tube insertion

In this pilot trial the aim was to evaluate a modified OSATS for chest tube insertion (see Table 1 (Tab. 1)). In depicting different levels of skill and allowing for individual feedback based on subscore assessment, this OSATS is meant to provide a valid tool for medical training and teaching. The modified OSATS tool for chest drain insertion was developed based on key steps of correct chest tube insertion, originally published by Hutton et al., which were modified and amended by a team of trauma and general surgeons [17]. Ten representative procedural steps can be evaluated separately using a 5-Point Likert scale complemented by detailed formulation of performance levels. Trainee’s performance in substeps adds up to a maximum total score of 50 points and a minimum score of 10 points, respectively [18]. 

The pilot trial was carried out in the surgical training center at the Department of General, Visceral, and Transplantation Surgery at Heidelberg University Hospital. Participants included in this study were medical students during their clinical years (3^rd^-6^th^ year) at Heidelberg University (n=9) as well as junior residents (1^st^ to 3^rd^ year, n=12), senior residents (4^th^ to 6^th^ year, n=14) and attending surgeons (n=6) from Heidelberg University Hospital. The pilot trial for evaluation of the modified OSATS score was conducted in January 2017. Chest tube insertions were performed on a cadaveric porcine model, which was prepared in a standardized way on an operating table in the supine position. At the beginning of the study, a brief introduction to training and assessment was held for each participant. Training started after the participant’s consent was obtained and pseudonymization had taken place. Each participant was asked to perform an initial training session using a mobile training device and the validated surgical training app Touch Surgery™ (TS) (Kinosis Ltd., London, UK) [15], [19]. The TS-module “Chest Tube Insertion” served as both an initial training to this intervention and a compact recapitulation for versed participants, respectively. Subsequent to app-based training, each participant was asked to perform a chest tube insertion on the porcine model using the provided instruments. Assessment was performed on the OSATS sheet by two independent and blinded raters via videos taped during the intervention. All videos were taped in a standardized way. To ensure blinding of raters the recorded image showed only the porcine thorax as well as the hands of the participants. Raters were specifically trained surgeons of the Department of General, Visceral, and Transplantation Surgery as well as the Center for Orthopedics, Trauma Surgery and Spinal Cord Injury of Heidelberg University Hospital. The final score of each trainee was calculated as an average score of the two independent ratings.

#### Ethics, consent and permissions

All data for the pilot study were recorded anonymously, treated confidentially, and were evaluated by authorized staff for scientific purposes only. Participants’ names were kept separate from all study data and were not used for the study. Each participant was assigned a designated code that was used for the entire study documentation and data collection. Participation in the study was voluntary. There were no foreseeable negative consequences for participants related to participation. The participating staff of the Heidelberg surgical center is experienced in the handling of animal models and training devices. Participants would have been excluded from the study in the event that a participant’s physical or mental health had become jeopardized due to participation in the present study. Ethical approval was obtained by the local ethics committee at Heidelberg University (A 13/14). Written informed consent was obtained from each participant.

#### Statistical analysis

Data collection was carried out by MS Excel^®^ 2016 (Microsoft^®^). Statistical analysis was carried out by SPSS Statistics Version 24.0 (IBM^®^ Germany). For statistical analysis, mean and standard deviation in case of continuous data, with absolute and relative frequencies for categorical parameters were used to describe the distributions of all parameters of interest. For OSATS scores, no Gaussian distribution was assumed, thus Kruskal-Wallis test was used as nonparametric test to compare all 4 groups. Moreover, Intraclass Correlation Coefficient was analyzed between the two video raters. For all tests, a p-value less than 0,05 was considered statistically significant. Where found to be appropriate, graphical statistical methods were deployed to illustrate findings.

## Results

### Results of the pilot study for evaluation of the objective structured assessment of technical skills for chest tube insertion

**Figure 1:**
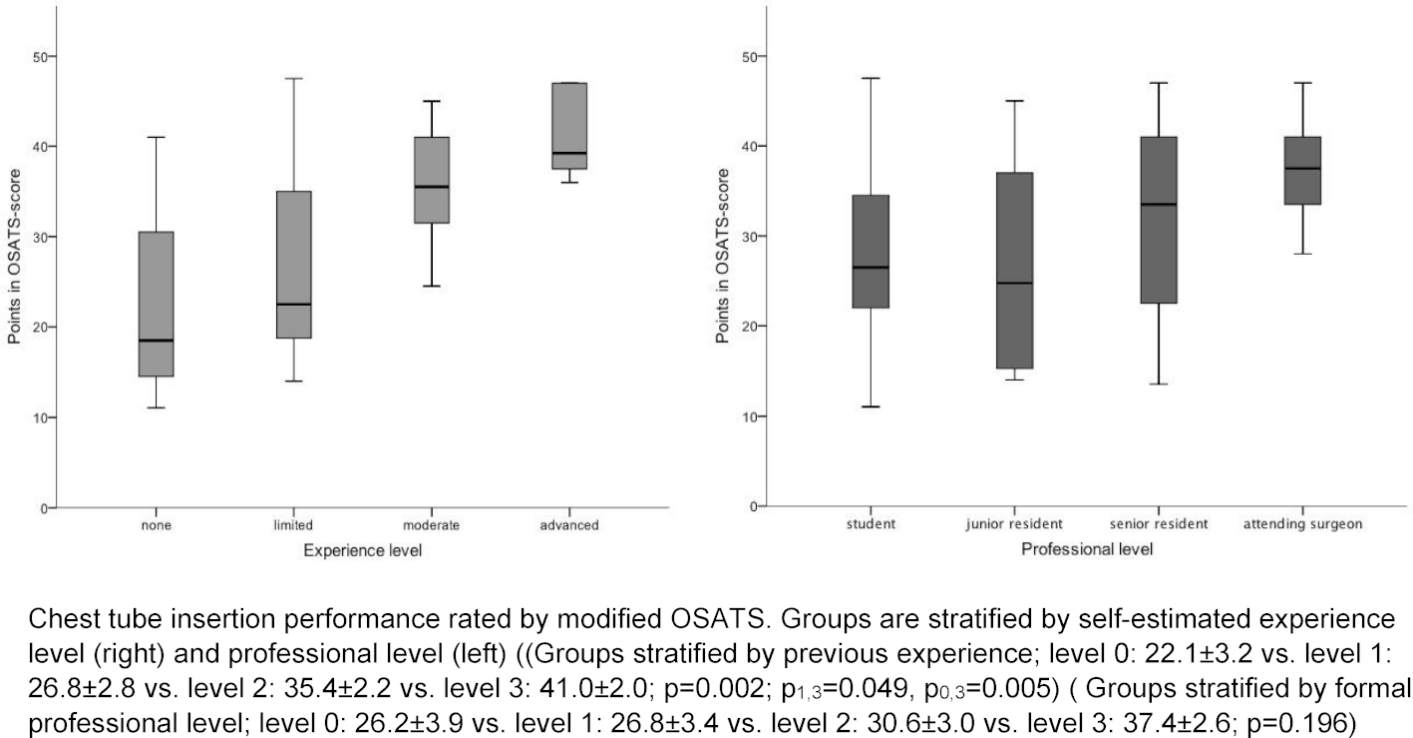
Results of the pilot study

A total of 41 participants were included in the pilot study (students=9; junior residents=12; senior residents=14; attending surgeons=6). Groups were stratified according to formal professional level (0=student, 1=junior resident, 2=senior resident, 3=attending surgeon) or self-estimated level of experience (0=none, 1=limited, 2=moderate, 3=advanced) There were significant differences between means of scores between four groups stratified by previous experience in chest tube insertion (level 0: 22.1±3.2 vs. level 1: 26.8±2.8 vs. level 2: 35.4±2.2 vs. level 3: 41.0±2.0) (see Table 2 (Tab. 2) and Figure 1 (Fig. 1)). Moreover, Kruskal-Wallis test revealed significant differences of group medians (p=0.002, Kruskal-Wallis statistic 14.6; Results of Dunn-Bonferroni-Test: p_0,3_=0.005, p_1,3_=0.049) assuming non-Gaussian distribution. However, if groups were stratified by formal professional level, no statistically significant distinction could be made using OSATS (level 0: 26.2±3.9 vs. level 1: 26.8±3.4 vs. level 2: 30.6±3.0 vs. level 3: 37.4±2.6) (see Table 2 (Tab. 2) and Figure 1 (Fig. 1)). Here Kruskal-Wallis-test showed a non-significant p-value of 0.196). Furthermore, analysis of the Intraclass Correlation Coefficient between the two independent expert raters showed an excellent agreement between the two ratings (ICC=0.96, 95% CI 0.91-0.98) [20].

## Conclusion

In the current pilot study, we evaluated the modified OSATS for chest tube insertion regarding it’s criterion validity for its use in blinded video rating. Video rating, instead of on-site direct rating, was used due to its ability to eliminate confounding factors such as age or formal professional level of the participants. Our results suggest that the OSATS was apt to standardize expert rating and could be used to depict and measure differences in skill between experience levels of surgeons, residents, and medical students. According to our results the OSATS tool is not biased by confounding factors like age or formal professional level, but reflects procedural experience only. 

Frameworks are essential for modern medical students education [4], [5], [6], [7]. As claimed by David et al., modern curricula need to be standardized and internationally comparable [7]. This need does not only exist for chest tube insertion training. In other medical disciplines, e.g. laparoscopic surgery, there is also a demand for a multimodal training frameworks, which also needs feedback via standardized and objective scores [21]. The comparability of learning success can be achieved through scores like the here proposed OSATS. Based on our results, the OSATS for chest tube insertion might be an instrument which can fulfill David et al.’s claims [7]. Moreover, concerning the needs of modern medical education, we believe this approach of assessment and feedback to prove additionally beneficial to outcome when teaching today’s medical trainees [16]. Moreover, we suggest that learning and teaching with the presented OSATS could be an answer to the needs of modern medical education as it offers clear defined learning goals combined with structured and objective assessment and feedback [7]. Furthermore, it offers a readily available and cost-efficient assessment tool for training of chest tube insertion. Our idea is that participants will profit from both the technical instructions from the OSATS as well as from feedback because their improvement in skill level can be subsequently objectified by the modified OSATS tool. Therefore, the OSATS might help to maximize patients’ safety. Also, this study will help to investigate potential improvements of current training curricula by the use of feedback and may even provide a reliable assessment for procedural proficiency certification for medical trainees with the modified OSATS aiming at a safe execution of this crucial emergency procedure. The OSATS can also be used in the context of certification processes as well as entrustable professional activities [7]. We consequently aim to use this scoring tool in courses for general surgery, trauma surgery and intensive care offered to surgical residents, trauma surgeons, anaesthesiologists, and medical students. After further investigation, the present OSATS might be integrated into modern curricula as part of competency based teaching programs. 

Nevertheless, some limitations of this pilot study should be noted. On the one hand this pilot study was conducted as single-center study at Heidelberg University Hospital. For this reason, the results of the current study are based on analyses with relatively small subgroups. On the other hand, the allocation of the participants to the four different subgroups, regarding their previous experience in using chest tubes, was based on the subjective self-evaluation of the participants. It is possible that there were inaccuracies between the subgroups due to under- or overestimation of the participants’ self-assessment. Further investigations and outcomes of the proposed study will increase the available knowledge about criteria to be met in order to ensure optimal surgical training – not only for trauma situations. 

## List of abbreviations

- OSATS: Objective Structured Assessment of Technical Skills
- TS: Touch Surgery
- ICC: Intraclass Correlation Coefficient

## Authors

- **Authors contributed equally:** Mirco Friedrich and Julian Ober
- **Study conception and design: **Nickel, Bergdolt, Ober, Friedrich, Haubruck, Müller-Stich, Tanner
- **Acquisition of data: **Nickel, Ober, Friedrich, Kowalewski, Bergdolt
- **Statistical analysis: **Ober, Bruckner
- **Analysis and interpretation of data:** Ober, Friedrich, Nickel, Bergdolt, Tanner, Haubruck, Müller-Stich
- **Drafting of manuscript:** Friedrich, Ober, Nickel, Kowalewski, Bergdolt, Bruckner
- **Critical revision: **Müller-Stich, Tanner, Haubruck

## Funding

The study was supported by the Heidelberg Surgery Foundation and the European Social Fund of the State Baden Wuerttemberg. 

## Competing interests

The authors declare that they have no competing interests.

## Figures and Tables

**Table 1:**
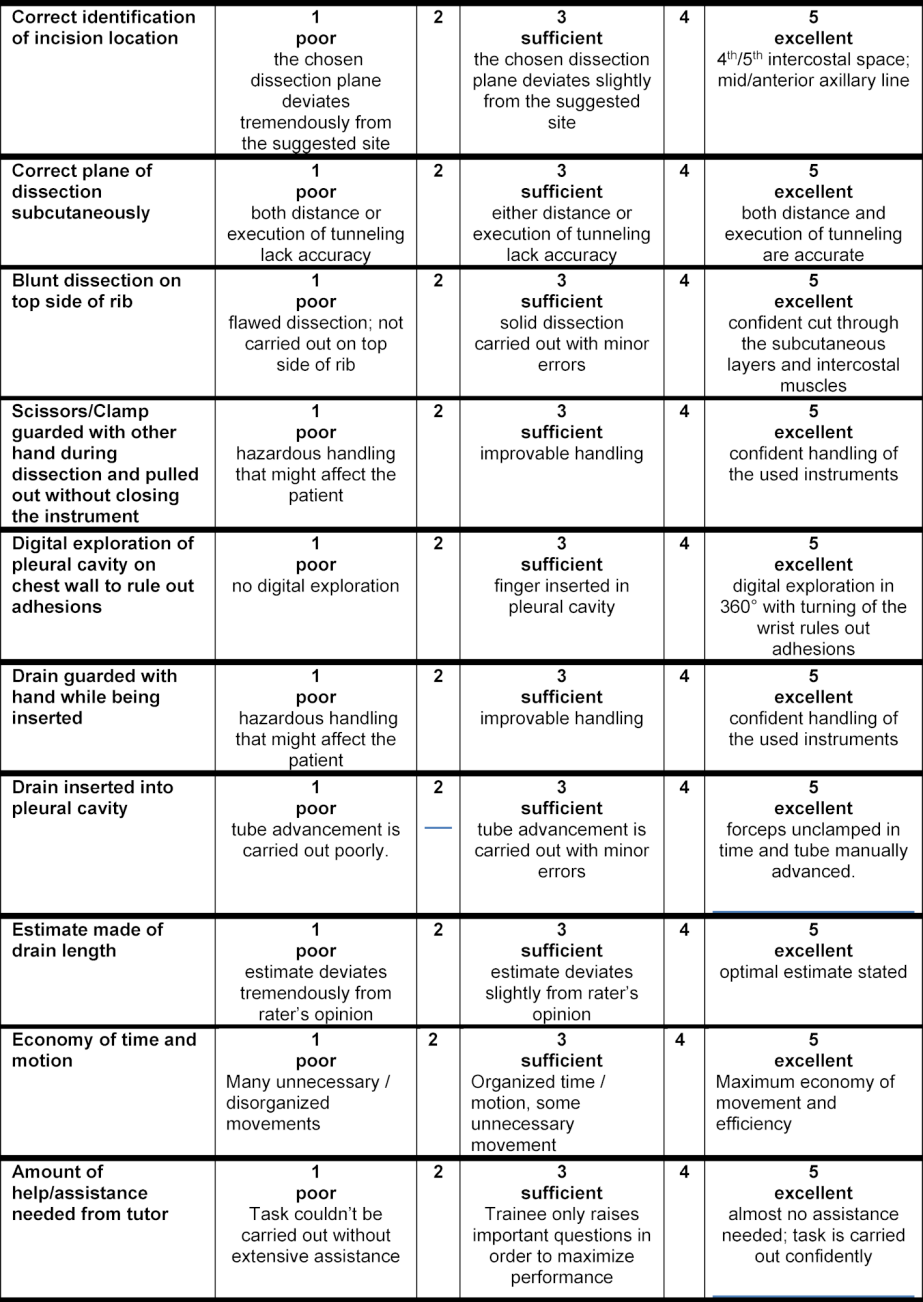
Objective structured assessment of technical skills score for chest tube insertion

**Table 2:**
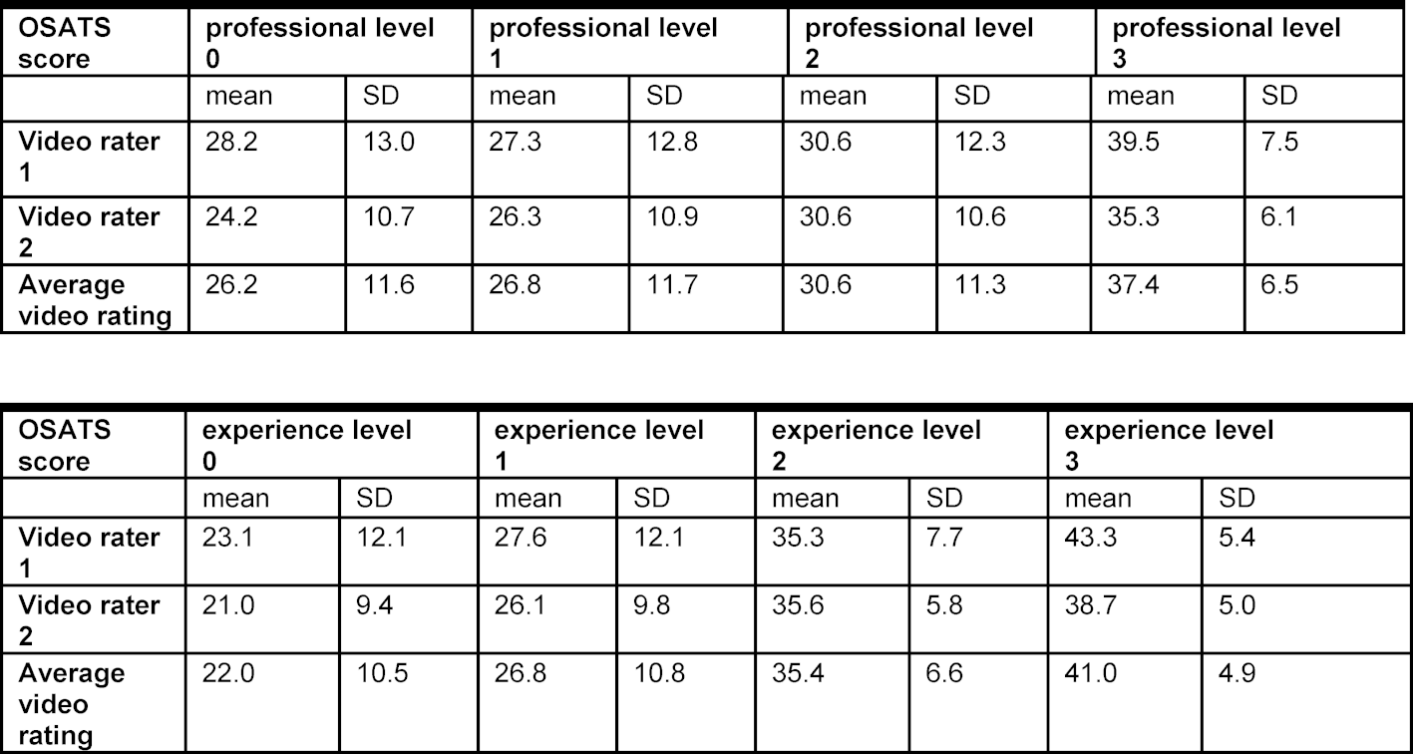
Results of the pilot study

## References

[1] Adler G, v. d. Knesebeck JH (2011). Bundesgesundheitsblatt Gesundheitsforschung Gesundheitsschutz.

[2] Kasch R, Engelhardt M, Forch M, Merk H, Walcher F, Frohlich S (2016). Zentralbl Chir.

[3] Carter BN (1952). The fruition of Halsted's concept of surgical training. Surgery.

[4] Lomis K, Amiel JM, Ryan MS, Esposito K, Green M, Stagnaro-Green A, Bull J, Mejicano GC, AAMC Core EPAs for Entering Residency Pilot Team (2017). Implementing an Entrustable Professional Activities Framework in Undergraduate Medical Education: Early Lessons From the AAMC Core Entrustable Professional Activities for Entering Residency Pilot. Acad Med.

[5] Brody H, Doukas D (2014). Professionalism: a framework to guide medical education. Med Educ.

[6] Irby DM, Hamstra SJ (2016). Parting the Clouds: Three Professionalism Frameworks in Medical Education. Acad Med.

[7] David DM, Euteneier A, Fischer MR, Hahn EG, Johannink J, Kulike K, Lauch R, Lindhorst E, Noll-Hussong M, Pinilla S, Weih M, Wennekes V (2013). The future of graduate medical education in Germany - position paper of the Committee on Graduate Medical Education of the Society for Medical Education (GMA). GMS Z Med Ausbild.

[8] Napolitano LM, Biester TW, Jurkovich GJ, Buyske J, Malangoni MA, Lewis FR Jr, Members of the Trauma, Burns and Critical Care Board of the American Board of Surgery (2016). General surgery resident rotations in surgical critical care, trauma, and burns: what is optimal for residency training?. Am J Surg.

[9] Roach PB, Roggin KK, Selkov G Jr, Posner MC, Silverstein JC (2009). Continuous, data-rich appraisal of surgical trainees' operative abilities: a novel approach for measuring performance and providing feedback. J Surg Educ.

[10] van Hove PD, Tuijthof GJ, Verdaasdonk EG, Stassen LP, Dankelman J (2010). Objective assessment of technical surgical skills. Br J Surg.

[11] Datta V, Bann S, Mandalia M, Darzi A (2006). The surgical efficiency score: a feasible, reliable, and valid method of skills assessment. Am J Surg.

[12] Aghdasi N, Bly R, White LW, Hannaford B, Moe K, Lendvay TS (2015). Crowd-sourced assessment of surgical skills in cricothyrotomy procedure. J Surg Res.

[13] Weitz G, Twesten C, Hoppmann J, Lau M, Bonnemeier H, Lehnert H (2012). Differences between students and physicians in their entitlement towards procedural skills education--a needs assessment of skills training in internal medicine. GMS Z Med Ausbild.

[14] Menger R, Telford G, Kim P, Bergey MR, Foreman J, Sarani B, Pascual J, Reilly P, Schwab CW, Sims CA (2012). Complications following thoracic trauma managed with tube thoracostomy. Injury.

[15] Pape-Koehler C, Immenroth M, Sauerland S, Lefering R, Lindlohr C, Toaspern J, Heiss M (2013). Multimedia-based training on Internet platforms improves surgical performance: a randomized controlled trial. Surg Endosc.

[16] Faulkner H, Regehr G, Martin J, Reznick R (1996). Validation of an objective structured assessment of technical skill for surgical residents. Acad Med.

[17] Hutton IA, Kenealy H, Wong C (2008). Using simulation models to teach junior doctors how to insert chest tubes: a brief and effective teaching module. Intern Med J.

[18] Friedrich M, Bergdolt C, Haubruck P, Bruckner T, Kowalewski KF, Muller-Stich BP, Tanner MC, Nickel F (2017). App-based serious gaming for training of chest tube insertion: study protocol for a randomized controlled trial. Trials.

[19] Kowalewski KF, Hendrie JD, Schmidt MW, Proctor T, Paul S, Garrow CR, Kenngott HG, Müller-Stich BP, Nickel F (2017). Validation of the mobile serious game application Touch Surgery for cognitive training and assessment of laparoscopic cholecystectomy. Surg Endosc.

[20] Koo TK, Li MY (2016). A Guideline of Selecting and Reporting Intraclass Correlation Coefficients for Reliability Research. J Chiropr Med.

[21] Nickel F, Kowalewski KF, Muller-Stich BP (2015). Chirurg.

